# Extraction of a Broken Needle from the Hand

**Published:** 1846-02-01

**Authors:** Estes Howe

**Affiliations:** From the Boston Medical and Surgical Journal


*Extraction of a broken Needle from the Hand—by Estes Howe, M. D. From the Boston Medical and Surgical Journal.*—There are few things more embarrassing, in ordinary practice, than being called upon for advice, in a case where a needle has been thrust into the flesh, and is supposed to be still present. Very often the evidence of its presence is so equivocal, that one feels very doubtful as to the expediency of an exploration with the scalpel, even at the request of the patient, and still more so as to advising or urging the use of the knife. Yet where a needle is really present, in the hand or foot, or near an articulation, the importance of immediate extraction will not be denied. And where there is no reasonable doubt of the presence of a needle, its exact position, and the direction of its axis, are extremely difficult to determine. We are not at liberty to explore, by incisions in every direction, as we might on the subject, but must content ourselves, at most, with a moderate crucial incision. and it is too often the case that after a search for some minutes, unsuccessfully, our conviction of the existence of the object of our search, "oozes out at the end of our fingers," like Bob Acre's courage—the patient "is sure it's not there," or "it feels better and may work out,"—while our back aches, our eyes are dim with looking, our fingers are tired of *poking*, and we give it up, in a woful uncertainty as to the case, but quite sure that the patient has an ugly wound to no purpose. To be assured, therefore, of the presence of a needle, and within very small limits, of its exact seat, and the direction of its axis, is no small thing in such cases. Feeling confidence in our diagnosis, we may boldly continue our explorations to complete success—being always able to assure our patient, beyond a doubt, that we shall ultimately succeed.

Case.—Mrs. F., while washing, thrust into the palmar surface of the right hand, about an inch and a half anterior to the pisiform bone, something sharp—probably a needle. Upon examination of the dress she was washing, the half of a needle was found, and the question was, whether the other half was, or was not, buried in the flesh. On examining the place, a small puncture was perceived, from which, I was told, no blood had issued, when I saw the patient an hour after the accident. I probed the puncture with the blunt end of a needle, pretty deeply, but could feel nothing, and upon pressure in various directions could not arrive at any unequivocal evidence of the presence of the broken portion of needle. The patient was very reluctant to submit to the scalpel, and I did not feel sufficiently sure to urge her to submit to it, while I was equally unwilling to have her run the risk of losing the usefulness of her right hand (upon which she and four children depended for bread,) by suffering the needle to remain, if it were really there. At this moment, the experiment of Mr. Alfred Smee, described in an article in the "Medical Times," of London, occurred to me, and I resorted to it with perfect satisfaction. His plan is to ascertain the existence and position of the needle, by rendering it a magnet. This may very readily be done, by subjecting it for a certain length of time to the action of a moderately powerful magnet. I procured, from a friend, a pretty powerful magnet—a steel bar about a foot long and a half inch square—well charged. This I bound upon the arm, placing one pole directly over the seat of the injury. Two hours after, I removed it, and upon bringing a small magnetic needle, about an inch and a half long, into the immediate vicinity of the injured part, had the great satisfaction of perceiving that it was strongly acted upon, being attracted or repelled as I presented one or the other pole. By a few experiments I was able to satisfy myself very nearly of the position of one pole of the magnet; but the exact direction of the axis I was not able to determine without an experiment that I could not well perform with a needle suspended in the ordinary way, upon a point. I therefore magnetized a common sewing needle, and suspended it by a fine silk thread. Upon bringing the affected part very near it, it was obviously influenced, and upon repeated trials uniformly arranged itself in a particular direction, of course parallel to the axis of the imbeded needle. I had now established the presence of the needle beyond all doubt, and its precise position and direction. A very moderate crucial incision enabled me to reach and extract it, though not without some trouble from the extreme timidity, and intolerance of pain, in the patient. I am sure that I could not have induced her to submit to the operation, unless I had had perfect confidence myself in my dagnosis. Perhaps it may be thought that the magnetic needle would have been attracted by the imbedded needle before it was magnetized, but I ascertained satisfactorily, by experiment, that this was not the case.

It is true, so large a magnet is not always at hand, but a smaller one would have been effectual, and any person possessing an electro-magnetic apparatus might make one of any size. I have been so much pleased with the result in this case, that I shall never use the knife, where I have any doubts, until I have cleared them up in the manner described.

